# Multifocal Extramedullary Plasmacytoma of the Thyroid With Cervical and Paratracheal Lymph Node Involvement and Progression to Multiple Myeloma

**DOI:** 10.7759/cureus.58847

**Published:** 2024-04-23

**Authors:** Vincent S Alexander, Michael D Ernst, Andrew D Vogel, Wang L Cheung, Alyssa N Obermiller, Said Baidas, Kavita M Pattani

**Affiliations:** 1 Department of Research, Alabama College of Osteopathic Medicine, Dothan, USA; 2 Department of Head and Neck Surgery, Orlando Health Cancer Institute, Orlando, USA; 3 Department of Pathology, Orlando Health Cancer Institute, Orlando, USA; 4 Division of Hematology/Oncology, Orlando Regional Medical Center, Orlando, USA

**Keywords:** thyroid pathology, extra-medullary plasmacytoma, cd138, mum1, extramedullary multiple myeloma

## Abstract

Extramedullary plasmacytomas without evidence of systemic illness make up less than 5% of all plasma cell neoplasms. The incidence of extramedullary plasmacytoma of the thyroid region is exceedingly rare. This report discusses the case of a 72-year-old male with extramedullary plasmacytoma of the thyroid. The patient underwent a total thyroidectomy for an enlarging right-sided thyroid nodule, and intraoperatively, the plasmacytoma was found to have an extracapsular component with adherence to the regional soft tissue as well as involvement of the right laryngeal nerve and regional lymph nodes. Despite a comprehensive negative workup for multiple myeloma initially, including a bone marrow biopsy and hematologic workup, the disease progressed to multiple myeloma following definitive radiation therapy, as evidenced by the development of hypermetabolic lytic lesions and further pathological examination. The patient’s treatment course included systemic chemotherapy and an autologous stem cell transplant, resulting in a favorable treatment response. The progression to multiple myeloma despite established guidelines highlights the need for close observation and the potential for innovative therapeutic strategies to manage this rare entity.

## Introduction

Plasma cell neoplasms are rare head and neck tumors, accounting for less than 1% of all head and neck tumors [1]. These masses have complex sequelae, given their proximity to vital anatomy, with this patient initially presenting with dysphagia. These neoplasms are generally classified based on the number of lesions involved. A solitary plasmacytoma represents a plasma cell neoplasm with a single lesion. Solitary plasmacytomas are further classified based on the location of the lesion. Most plasmacytomas are located within the bone and are termed solitary bone plasmacytomas, also known as intramedullary plasmacytomas [2].

Plasmacytomas located in soft tissue are referred to as extramedullary plasmacytomas and are rarer than intramedullary plasmacytomas [2]. Extramedullary plasmacytomas comprise only 3%-5% of all plasma cell neoplasms [3]. Plasma cell neoplasms involving multiple lesions are usually called multiple solitary plasmacytomas. A secondary concern for patients diagnosed with intramedullary or extramedullary plasmacytomas is progression towards multiple myeloma. Within 10 years, 32.7% of patients diagnosed with an extramedullary plasmacytoma progress to multiple myeloma [4]. Treatment modalities are usually systemic in nature, with chemotherapy being the primary modality. This case report presents a multifocal extramedullary plasmacytoma of the thyroid with extracapsular extension and nerve involvement, which ultimately progressed to multiple myeloma.

## Case presentation

A 72-year-old male presented with a primary complaint of dysphagia as well as a central neck mass. Thyroid ultrasound revealed a 3.6 x 2.7 x 2.4 cm nodule of the right mid-thyroid. Ultrasound-guided fine needle aspiration of the nodule revealed a benign follicular nodule with no evidence of malignancy.

The patient underwent a total thyroidectomy. Intraoperatively, a large and calcified right thyroid mass was adherent to the trachea, the esophageal musculature, and the overlying infrahyoid muscles wrapped around the right recurrent laryngeal nerve. The infrahyoid muscles overlying the mass were removed, and the esophageal and neck floor musculature, including scalenes and pharyngeal constrictors, were also dissected to show clear extrathyroidal extension.

**Figure 1:**
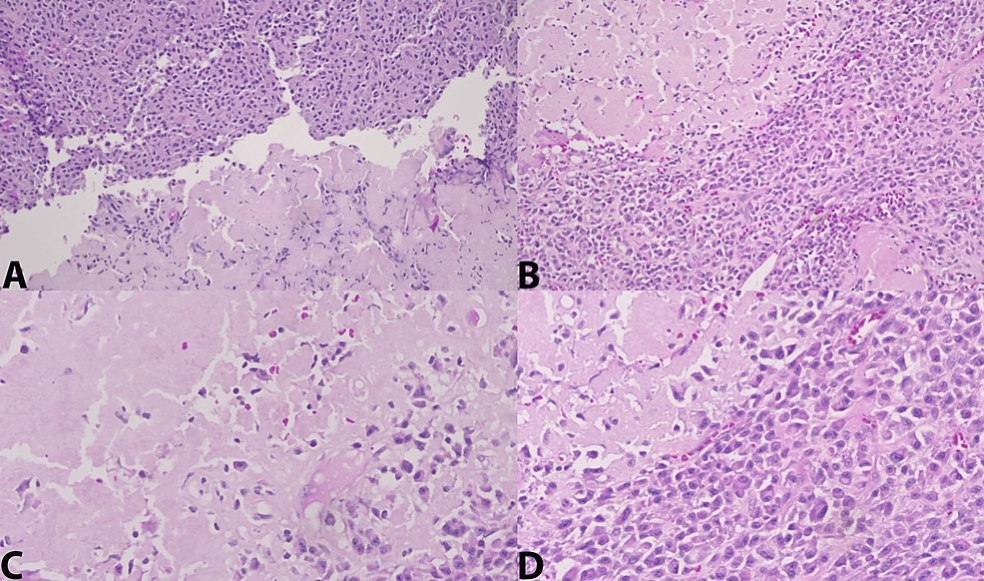
The sections of the mass, as seen in the cervical lymph node biopsy and dissection specimen, are composed of sheets of plasma cells (CD138 weakly positive) with extensive amyloid deposition. Given the extensive amyloid deposition and proximity to the thyroid gland, a medullary carcinoma was excluded by negative reactivity for thyroid transcription factor 1 (TTF-1), calcitonin, chromogranin, and monoclonal-carcinoembryonic antigen (CEA). The plasma cells are diffusely and strongly positive for multiple myeloma oncogene 1 (MUM1) with the expression of CD79a, lambda restriction, and null kappa. CD20 and CD3 highlight normal residual nodal architecture. A: Core biopsy H&E at 200x magnification. B: Lymph node dissection at 200x magnification. C: Lymph node dissection at 400x magnification with amyloid. D: Lymph node dissection at 400x magnification.

Final surgical pathology revealed plasma cell neoplasm with extensive amyloid deposition of the right thyroid lobe with involvement of a paratracheal lymph node. The tumor revealed solid sheets of cells that stained weakly positive for CD138 (syndecan-1). Staining for calcitonin, chromogranin, monoclonal-carcinoembryonic antigen (CEA), and thyroid transcription factor 1 (TTF-1) was negative. The plasma cells of the paratracheal node stained strongly positive for multiple myeloma oncogene 1 (MUM1) with a dim expression of CD79a, lambda restriction, and null kappa (Figure 1). Histologically, the contralateral lobe demonstrated normal thyroid parenchyma.

**Figure 2:**
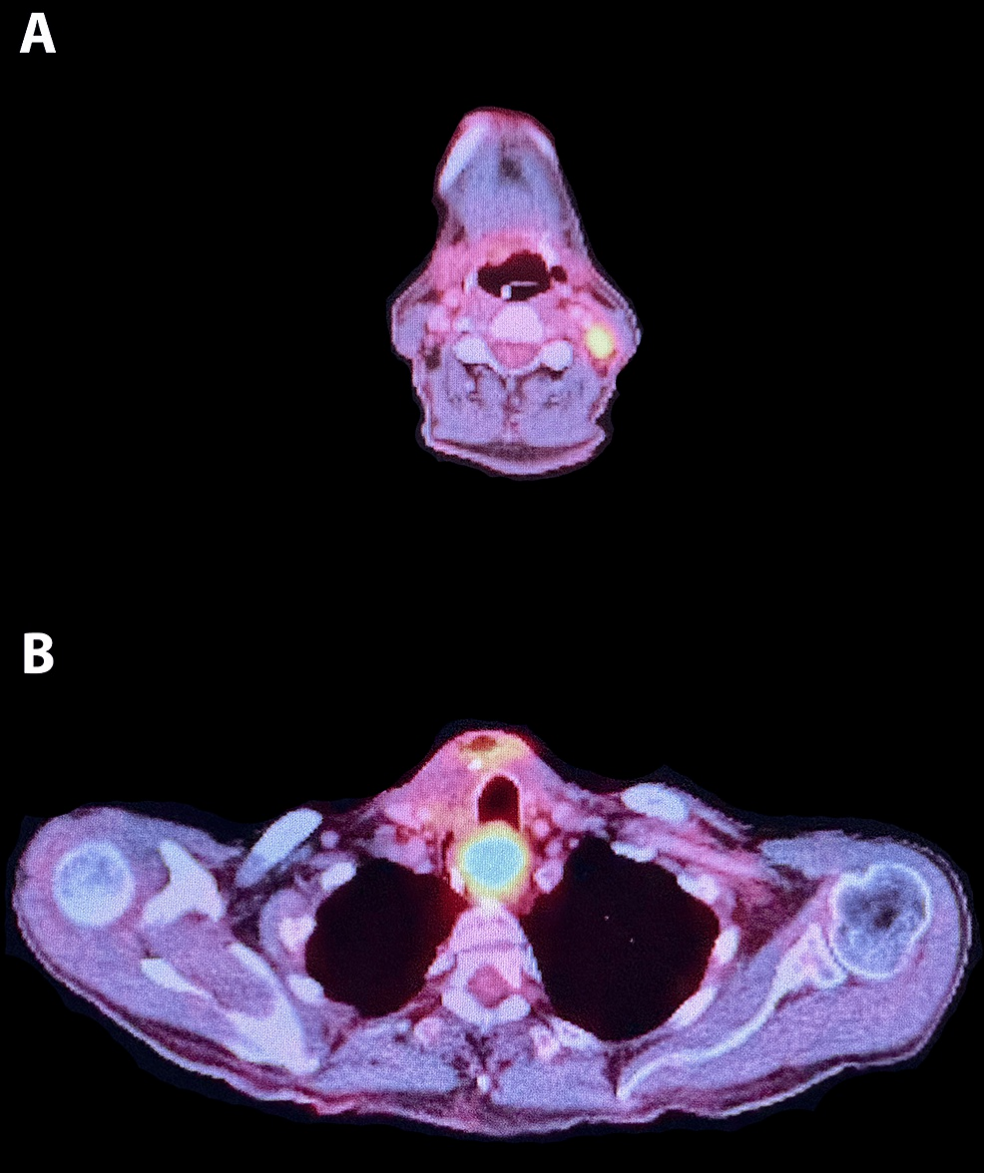
Post-operative positron emission tomography-computed tomography (PET/CT) scan. A: Axial image: hypermetabolic left level II cervical neck lymph node, highly worrisome for malignancy. B: Axial image: intensely hypermetabolic soft tissue mass in the proximal esophagus, highly worrisome for malignancy. Prominent metabolic activity in the thyroid surgical bed, favoring post-surgical inflammatory changes. Metabolically active right level II and IV cervical neck lymph nodes, the relatively mild diffuse nature of the activity, favoring reactive/inflammatory changes.

Post-operative positron emission tomography-computed tomography (PET/CT) (Figure 2) revealed a hypermetabolic left level II cervical neck lymph node and an intensely hypermetabolic soft tissue mass in the proximal esophagus, which suggests malignancy. Suspicious or reactive lymph nodes in the right level III and IV necks were also identified. The patient subsequently underwent an ultrasound-guided core needle biopsy (Figure 1: image A) of the left level II hypermetabolic node that revealed a plasma cell neoplasm consistent with previous thyroid pathology.

Bone marrow aspiration revealed normal cellular (30%) marrow with adequate trilineage hematopoiesis and no evidence of disease. There was no morphologic evidence of plasma cell neoplasm, and no monoclonal B-cell and T-cell populations were present. The percentage of blast cells identified was 3%. On hematologic workup, the patient’s immunoglobulin levels were IgA of 269 mg/dL, IgG of 1190 mg/dL, and IgM of 113 mg/dL. There was no M-spike on immunofixation. The patient’s beta-2 glycoprotein level was 2.57 U/mL. His serum-free light chain assay revealed a kappa level of 2.89 mg/L and a slightly elevated lambda level of 3.78 mg/L with a kappa/lambda ratio of 0.76. Further laboratory testing revealed a plasma calcium level of 9.3 mg/dL and a creatinine of 0.85 mg/dL. All lab results were within normal limits, and the patient was diagnosed with extramedullary plasmacytoma of the head and neck with no signs of multiple myeloma.

The patient completed intense modulated radiation therapy with image-guided therapy. The clinical target volume included the presurgical primary disease in the thyroid, the gross tumor within the bilateral cervical lymph nodes, and the paraesophageal mass.

Post-treatment PET/CT revealed interval development of multiple new hypermetabolic lytic lesions throughout the thoracic and abdominopelvic skeleton. The osseous lesions were located at L2, L3, L5, and S1. The patient underwent an IR-guided biopsy of the hypermetabolic L4 lesion and an additional bone marrow biopsy. The pathology from the bone marrow biopsy revealed normal cellular (40%) bone marrow with no atypical infiltrates. CD138 immunostaining identified 2% plasma cells. Plasma cells were polytypic, and no clonal B-cell population was identified with flow cytometry. Cytogenetics of this patient’s bone marrow sample demonstrated a normal male karyotype, and the fluorescence in situ hybridization (FISH) profile for multiple myeloma was negative. IR-guided biopsy of the L5 lesion was also performed and revealed bone marrow with trilineage hematopoiesis, which was negative for metastatic carcinoma or plasma cell neoplasm. Immunostaining for CD38 highlighted rare and scattered plasma cells with less than 5% cellularity.

**Figure 3:**
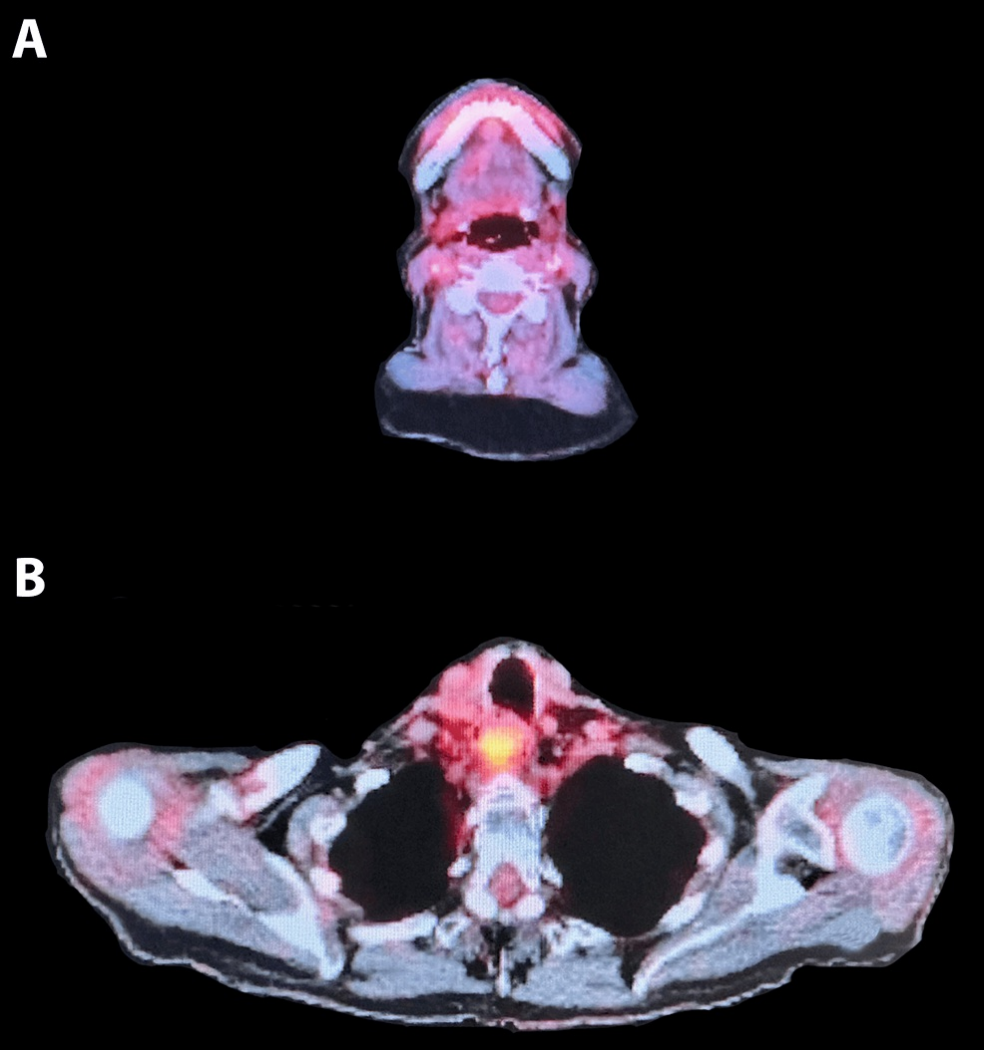
Post-chemotherapy and bone marrow transplant imaging: no definitive PET/CT evidence of malignancy. Metabolically active soft tissue fullness in the proximal esophagus is stable compared to the previous study. A: Axial image of the previously metabolic level II node. B: Axial image of previous metabolic esophageal mass.

Given the appearance of multiple osseous lesions on post-radiation imaging, the patient was diagnosed with multiple myeloma and underwent systemic chemotherapy with lenalidomide, bortezomib, and dexamethasone (RVD). Post-treatment PET/CT revealed resolution of previously seen hypermetabolic osseous activity consistent with excellent therapeutic response (Figure 3). The patient subsequently underwent an autologous stem cell transplant and is currently without evidence of disease.

## Discussion

Extramedullary plasmacytomas are non-specific to soft tissue but are found to be prevalent in the head, neck, and upper respiratory tract [5]. The thyroid is rarely the primary site [6]. There have been less than 31 documented cases of extramedullary plasmacytomas of the thyroid since 1994 [7]. There is one case report of cervical lymph node involvement [1]. However, given the extensiveness of the disease in this case, with nerve extension and multiple sites of nodal involvement, there is a lack of evidence-based guidelines for appropriate clinical management. The average age-adjusted 5-year survival rate of 21 patients with extramedullary plasmacytomas of the thyroid was found to be 73.2% [8]. However, given the limited sample size, the data’s significance is limited.

This patient’s progression from multiple extramedullary plasmacytomas to multiple myeloma further highlights the need to identify effective therapeutic options to prevent disease progression in patients with multiple plasmacytomas. The oncogene MUM1 was strongly expressed in this patient’s case and is known to activate the promoter for the monokine induced by the interferon-gamma (MIG) gene within B-cells, thus promoting oncogenesis and progression to B-cell malignancies such as multiple myeloma and B-cell lymphoma. The interaction between the MUM1 and MIG genes has been studied as a potential therapeutic target to halt oncogenesis with antibodies against MIG, limiting the proliferation of MUM1-expressing cell lines [9,10]. The strong expression of MUM1 in this patient points to the potential for future studies to identify a possible therapy targeted at this MUM1/MIG relationship to prevent progression from multiple plasmacytomas to multiple myeloma. New treatment modalities involving chimeric antigen receptor T-cell therapy (CAR-T) are also currently being studied and have been shown to reduce tumor burden in one patient with a refractory orbital plasmacytoma [11].

Superior outcomes with extramedullary plasmacytoma for patients with head and neck primary sites have been discussed, with superior 5-year overall survival and disease-specific survival rates compared with non-head and neck extramedullary plasmacytomas [6]. This same study also found that head and neck plasmacytomas are more responsive to surgery with or without radiation versus radiation alone. This pathophysiologic mechanism has not yet been established [6].

This patient did not meet the current diagnostic criteria for multiple myeloma and was refractory to radiotherapy. Plasmacytomas have been found to be sensitive to radiotherapy [12]. However, in this case, the primary tumor was not sensitive to radiotherapy, and the patient subsequently developed multiple myeloma, suggesting that the current delineation between plasmacytoma and multiple myeloma might leave unexpected gaps for rare cases that do not classically fit multiple myeloma criteria but might benefit from treatment modalities specific to multiple myeloma.

## Conclusions

This case of multifocal extramedullary plasmacytoma of the thyroid, subsequently progressing to multiple myeloma, highlights the intricate nature of plasma cell neoplasms and the need for close monitoring for potential progression in this specific cohort. It elucidates a rare case of extrathyroidal extension into soft tissue and highlights the challenges of managing such unique presentations. The progression of the disease into multiple myeloma highlights, despite aggressive therapy, calls for further study into tailored management strategies and the potential development of more refined guidelines to enhance patient outcomes and survival in these rare and complex cases.

## References

[1] Ridal M, Ouattassi N, Harmouch T, Amarti A, Alami MN (2012). Solitary extramedullary plasmacytoma of the thyroid gland. Case Rep Otolaryngol.

[2] Dimopoulos M, Terpos E, Comenzo RL (2009). International myeloma working group consensus statement and guidelines regarding the current role of imaging techniques in the diagnosis and monitoring of multiple myeloma. Leukemia.

[3] Hassan MJ, Khans S, Pujani M, Jetley S, Raina PK, Ahmad R (2014). Extramedullary plasmacytoma of the thyroid: report of a rare case. Blood Res.

[4] Thumallapally N, Meshref A, Mousa M, Terjanian T (2017). Solitary plasmacytoma: population-based analysis of survival trends and effect of various treatment modalities in the USA. BMC Cancer.

[5] Alexiou C, Kau RJ, Dietzfelbinger H (1999). Extramedullary plasmacytoma: tumor occurrence and therapeutic concepts. Cancer.

[6] Gerry D, Lentsch EJ (2013). Epidemiologic evidence of superior outcomes for extramedullary plasmacytoma of the head and neck. Otolaryngol Head Neck Surg.

[7] Refai F, Gomaa W, Abdullah L (2020). A case report of thyroid plasmacytoma and literature update. J Microsc Ultrastruct.

[8] Dores GM, Landgren O, McGlynn KA, Curtis RE, Linet MS, Devesa SS (2009). Plasmacytoma of bone, extramedullary plasmacytoma, and multiple myeloma: incidence and survival in the United States, 1992-2004. Br J Haematol.

[9] Uranishi M, Iida S, Sanda T (2005). Multiple myeloma oncogene 1 (MUM1)/interferon regulatory factor 4 (IRF4) upregulates monokine induced by interferon-gamma (MIG) gene expression in B-cell malignancy. Leukemia.

[10] Tsuboi K, Iida S, Inagaki H (2000). MUM1/IRF4 expression as a frequent event in mature lymphoid malignancies. Leukemia.

[11] Lin RZ, Lu T, Homer N, Men CJ (2024). Successful treatment of refractory orbital plasmacytoma with chimeric antigen receptor t cell therapy: a case report and review of the literature. Ophthalmic Plast Reconstr Surg.

[12] Galieni P, Cavo M, Pulsoni A (2000). Clinical outcome of extramedullary plasmacytoma. Haematologica.

